# Topic Issue: “Translational Advances in Neurodegenerative Dementias”

**DOI:** 10.3390/neurolint17020031

**Published:** 2025-02-19

**Authors:** Annibale Antonioni, Francesco Di Lorenzo

**Affiliations:** 1 Doctoral Program in Translational Neurosciences and Neurotechnologies, Department of Neuroscience and Rehabilitation, University of Ferrara, 44121 Ferrara, Italy; annibaleantonioni658@gmail.com; 2 Experimental Neuropsychophysiology Lab, Department of Clinical and Behavioural Neurology, Santa Lucia Foundation IRCCS, 00179 Rome, Italy

Neurodegenerative dementia, a collective term for a range of disorders characterized by progressive cognitive and functional decline, is an urgent challenge in global healthcare [1,2,3]. Indeed, disorders such as Alzheimer’s disease (AD), frontotemporal dementia, and Lewy body dementia impose a severe burden on patients, caregivers, and healthcare systems [4,5,6]. Their heterogeneity underscores the critical need for multifaceted research approaches to develop innovative diagnostic and therapeutic strategies [7,8]. This Special Issue brings together contributions that address the complexity of neurodegenerative dementias from diverse perspectives, employing advanced methodologies to explore these disorders’ molecular and systemic dimensions.

Importantly, given their complex and heterogeneous pathophysiology, which remains only partially understood, animal models are indispensable for elucidating neurodegeneration mechanisms and identifying therapeutic targets [9,10,11,12]. In particular, since specific genetic mutations have a well-established pathogenetic role (e.g., *Presenilin 1* and *2* in AD), animal models allow the consequences of such alterations to be explored in-depth, recapitulating in a limited timeframe changes observed in humans after decades [13,14,15]. For example, Thompson et al. (Contribution 1) investigated the effects of three months of exposure to levetiracetam (i.e., a widely used anti-epileptic drug) in Tg4510 mice expressing mutant human Tau showed only modest effects on Tau pathology and behavior [16]. This contrasts with the promising results observed in amyloid deposition models and highlights significant efficacy differences depending on the experimental neuropathological model. Interestingly, Klimenko et al. (Contribution 2) investigated the impact of chronic sleep deprivation in a mouse model of familial AD, revealing consequences on cognitive performance and their neurobiological underpinnings. Similarly, Lee et al. (Contribution 3) evaluated the effects of maxillary malocclusion in a mouse model of vascular dementia, yielding comparable findings from biological and behavioral perspectives [17]. These aspects are particularly relevant, considering that complex relationships between sleep disturbances, temporomandibular alterations, and neurodegenerative processes have also been suggested in humans [18,19]. Identifying the neurobiological mechanisms involved in these processes might aid in developing targeted treatments and, more importantly, enhance education and preventive strategies to mitigate their onset [20,21]. Indeed, a deeper understanding of the pathophysiology of neurodegenerative diseases has paved the way for exploring carotenoids in this context. Their relevant antioxidant and anti-neuroinflammatory properties hold significant promise as therapeutic agents and preventive measures against the onset of neuropathological damage, as discussed by Gandla et al. (Contribution 4).

Moreover, Mohamed-Mohamed et al. (Contribution 5) provided essential insights into the pathophysiology of dementia in human patients as well, particularly with a comprehensive review of the link between type two diabetes mellitus, vascular dementia, and AD. These findings support the growing body of evidence highlighting lifestyle’s crucial role in preventing or slowing disease progression. Furthermore, intracranial compliance has been extensively examined by Gholampour (Contribution 6), underscoring its critical clinical significance in diagnosing and assessing treatment outcomes for brain disorders. This, in turn, plays a vital role in preventing the initiation of neurodegenerative processes. Similarly, as highlighted by Lin et al. (Contribution 7), vitamin D supplementation proved to be a significant preventive strategy in vulnerable populations, as it reduced the risk of dementia onset in a dose-dependent manner, regardless of age, in hemodialysis patients. These findings suggest that interventions aimed at counteracting neurodegenerative mechanisms can be applied in everyday life and the clinical setting, and it is hoped that this Special Issue will provide valuable insights among the general population and healthcare professionals. Importantly, blood-based biomarkers, such as phosphorylated Tau, have recently proven effective in non-invasively identifying patients with amyloid pathology regardless of cognitive status [22,23]. Such advancements could facilitate the early identification of patients eligible for preventive measures and enrollment in clinical trials, ultimately supporting timely intervention efforts such as those discussed here [24].

However, theoretical models about the evolution of symptoms are also crucial to understanding the pathophysiology of neurodegenerative dementias. In this regard, it is worth mentioning Pirani’s review (Contribution 8), which explores the concept of retrogenesis in dementias, highlighting how the loss of cognitive functions mirrors the reverse order of their developmental acquisition. Indeed, by tracing the progression from infantile “Anoetic Body Consciousness” to adult Autobiographical Memory and self-awareness, the author proposes that dementia-related cognitive decline follows a retrogenetic trajectory, culminating in a return to primitive body-centered functions, where the last functions to disappear are chewing/swallowing. The findings discussed underscore the relevance of a biopsychosocial, person-centered approach to understanding and managing dementia.

Further studies have also embraced highly innovative research areas, leveraging recent technological advances in neuroscience. Consistently, identifying biomarkers capable of reliably diagnosing and predicting disease prognosis remains a pivotal goal in various domains of neurology. For instance, measures derived from electroencephalography (EEG), noninvasive brain stimulation techniques (NIBS), and artificial intelligence (AI) methods have shown great promise in this context, with interest in their application rapidly growing [25,26,27,28,29]. Montoya-Pedrón et al. (Contribution 9), in particular, demonstrated the utility of an EEG biomarker, namely the total amplitudes of the contingent negative variation potential, i.e., a cognitive event-related potential evaluating attention and orienting responses, in distinguishing between normal cognitive functioning and neurocognitive disorders potentially due to AD [30]. Additionally, studies conducted by Cai et al. and Wang et al. (Contributions 10 and 11) employed AI techniques such as machine learning and dual semi-supervised learning to classify patients with mild cognitive impairment (MCI) and AD or to predict the progression of MCI to AD, respectively. This is particularly critical as applying such AI algorithms could extend findings observed in single studies to broader patient populations [31]. For instance, NIBS techniques demonstrated the ability to predict conversion to overt dementia, classify different MCI subtypes, and even provide meaningful therapeutic support in these contexts [32,33,34,35]. Consistently, by integrating AI methodologies with cutting-edge diagnostic tools, researchers and clinicians could significantly enhance their capacity to intervene early and tailor treatments, ultimately improving outcomes for individuals affected by these complex conditions. Therefore, technology will likely play an increasingly pivotal role in advancing the diagnosis and treatment of neurodegenerative diseases [36,37].

Collaborative efforts spanning basic science and technological advancements are essential to bridge these gaps and translate research findings into clinical breakthroughs. We extend our gratitude to the Authors for their valuable contributions and to the Reviewers for their rigorous evaluations, which have enriched the scientific quality of this Special Issue. It is our hope that the insights presented here will inspire future research endeavors and ultimately contribute to alleviating the burden of neurodegenerative dementias.

## Data Availability

Not applicable.
